# Exploring Trop-2–Nanobody PPI Interactions through Molecular Dynamics Simulations and Biovalidations

**DOI:** 10.1021/acsomega.5c11494

**Published:** 2026-03-05

**Authors:** Jin Cheng, Ze-Yu Sun, Zhiyuan Guo, Yixuan Hao, Gavin Hou, Yijin Li, Yuanqiang Wang, Zhiwei Feng, Ying Xue, Li Meng

**Affiliations:** † School of Pharmacy, 117898 Jiangsu Vocational College of Medicine, Yancheng 224005, P.R. China; ‡ Department of Pharmaceutical Sciences, Computational Chemical Genomics Screening Center, and Pharmacometrics & System Pharmacology PharmacoAnalytics, School of Pharmacy; National Center of Excellence for Computational Drug Abuse Research, 704566 University of Pittsburgh, Pittsburgh, Pennsylvania 15261, United States; § 612638 Yancheng No.1 People’s Hospital, Yancheng 224006, P.R. China; ∥ College of Engineering, 1259 University of Michigan Ann Arbor, Ann Arbor, Michigan 48109, United States; ⊥ School of Pharmacy and Bioengineering, 6614 Chongqing University of Technology, Chongqing 400054, P.R. China; # Faculty of Pharmaceutical Sciences, 12478 Shenzhen University of Advanced Technology, Shenzhen 518107, China; ∇ Department of Pharmacy, Zhongshan Hospital, Fudan University, Shanghai 200032, China

## Abstract

The transmembrane glycoprotein Trop-2 has garnered significant attention as a potential therapeutic target due to its involvement in various malignancies, including breast, lung, and prostate cancers. Specifically, the exploration of Trop-2-specific antibodies and nanobodies has emerged as a promising avenue for innovative treatment strategies. Despite advancements, a comprehensive understanding of the intricate molecular interactions between Trop-2 and specific nanobodies remains elusive. In this study, molecular dynamics (MD) simulations with MM/GBSA were employed to investigate the binding poses, key residues, and detailed interactions between Trop-2 and three distinct nanobodies (Nb60, Nb65, and Nb108), which will further guide the development of Trop-2-targeting nanobodies. Our findings corroborated the docking results, highlighting the reliability of binding pose 1. Additionally, our simulations elucidated key residues involved in the interaction interface, particularly within the C-terminal cysteine-poor domain (CPD) and an α-helix region (S170–Y185) on Trop-2. Furthermore, we identified the critical role of the complementarity-determining region 3 (CDR3) length and the key residues involved in the binding of nanobodies to Trop-2. Potential key residues predicted by MD simulations guided the redesign of the CDR3 region of Nb60, generating variants Nb-6, Nb-7, and Nb-14. Experimental data showed that alterations in the CDR3 significantly impacted binding affinity, with Nb-7 exhibiting the highest affinity (*K*_d_ value of 7.931 μM), whereas Nb-6 and Nb-14 showed reduced or no binding. This comprehensive analysis provides valuable insights into the molecular mechanisms governing Trop-2–nanobody interactions, facilitating future nanobody engineering efforts for targeted cancer therapy.

## Graphical abstract

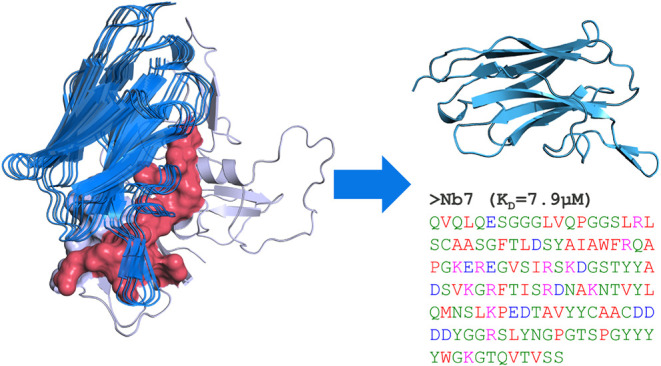

## Introduction

Trop-2, also referred to as tumor-associated calcium signal transducer 2 (TACSTD2),^1–6^ stands out as a pivotal transmembrane glycoprotein that intricately involved in a diverse range of physiological and pathological processes. Its structural complexity is underscored by an extracellular domain containing epidermal growth factor (EGF)-like repeats, a transmembrane domain, and a short cytoplasmic tail, all of which emphasize its multifaceted roles in cellular signaling, adhesion, and the modulation of growth factor responses.^3,5–9^

Extensive research has illuminated Trop-2’s pervasive presence across various cancer types, spanning breast, lung, colorectal, prostate, and pancreatic cancers, highlighting its pivotal involvement in oncogenesis and the progression of tumors.^10^ Moreover, beyond its established association with cancer, Trop-2 has been implicated in fundamental cellular functions such as proliferation, survival, migration, and adhesion, underscoring its indispensable role in maintaining tissue homeostasis and physiological balance.^11^

Dysregulation of Trop-2 expression has been closely linked to malignant transformation, characterized by aberrant cellular proliferation, evasion of apoptosis, heightened migration, and invasive properties.^12,13^ In cancer settings, Trop-2’s overexpression often aligns with advanced disease stages, dismal prognosis, and resistance to conventional therapies, positioning it as a promising diagnostic biomarker and an appealing therapeutic target for interventions aimed at halting disease progression and enhancing patient outcomes.^14–17^

In the pursuit of targeted therapeutic interventions for Trop-2-associated malignancies, researchers have explored diverse strategies, ranging from antibody-drug conjugate (ADC) to cutting-edge nanobody technology.^8,18–25^ For instance, the approval of the first Trop-2-targeted ADC, IMMU-132, in April 2020, has opened up new avenues for cancer treatment.^26,27^ MAAP-9001a, a humanized IgG1 antibody with moderate affinity for Trop-2 (*K*_d_ = 27 nM), was designed to expand the drug’s therapeutic window, and optimize both efficacy and safety. Findings from the TROPION-Lung01 study indicate that datopotamab deruxtecan (Dato-DXd) monotherapy enhances survival outcomes in patients with locally advanced or metastatic non‑small cell lung cancer (NSCLC), irrespective of their actionable genomic alteration (AGA) status.^28^ SKB264 is a novel ADC targeting Trop-2, incorporating the same antibody component as IMMU-132. Its payload is a derivative of belotecan, KL610023 (T030), a powerful topoisomerase I inhibitor, attached through an enzymatically cleavable linker to improve stability and minimize toxic side effects.^29,30^ In addition, recent advancements in nanobody engineering have introduced a promising alternative avenue for precision therapy. Nanobodies, derived from camelid antibodies, offer distinct advantages such as their smaller size, superior tissue penetration, enhanced stability, and adaptability in engineering. For example, recent strides^18^ in nanobody research have culminated in the development of Trop-2-specific nanobodies, notably Nb60, Nb65, and Nb108, which have exhibited remarkable affinity and specificity for Trop-2. Through meticulous engineering processes, including biopanning against immobilized recombinant Trop-2, these nanobodies have been honed to display potent antitumor activities, including the inhibition of cell proliferation and migration.

In our prior investigation, we enhanced the Molecular Complex Characterizing System (MCCS)^31–36^ specifically for studying protein–protein interactions, and utilized it to explore the molecular interactions between Trop-2 and three distinct nanobodies—Nb60, Nb65, and Nb108. We initiated homology modeling of the three nanobodies to derive their respective three-dimensional structures. Subsequently, we performed docking simulations to generate the nanobody receptor complex with Trop-2. Subsequent to this, we applied the MCCS protocol to pinpoint crucial amino acid residues and conducted an energy analysis, providing specific contribution values for the amino acid residues involved. Notably, our analysis unveiled that nanobodies with elongated Complementarity Determining Region 3 (CDR3) exhibited reduced binding energies, indicating enhanced affinity. Remarkably, this observation was consistent with experimental affinity data.

Building upon our previous work, in which we further refined MCCS to explore the molecular interplay between Trop-2 and nanobodies, the current study delved deeper into the dynamics of these interactions using molecular dynamics (MD) simulations.^36–39^ Specifically, we focused on elucidating the binding poses, key residues, and interactions between Trop-2 and the aforementioned nanobodies (Nb60, Nb65, and Nb108) to assist our design of novel nanobody‑based therapeutics. By validating previous docking results and favoring binding pose 1, our MD simulations provide additional insights into the reliability of molecular docking. The present work is to unravel the dynamic behavior of Trop-2–nanobody complexes, offering a comprehensive understanding of the molecular mechanisms governing their interactions.

This study contributes to a better understanding of Trop-2–nanobody interactions, furthering the development of targeted therapeutic strategies for Trop-2-associated malignancies. By delineating key residues and binding mechanisms, our findings contribute to the ongoing pursuit of precision medicine approaches aimed at combating cancer.

## Methods and Materials

### Homology Modeling Using SWISS-MODEL

SWISS-MODEL^40^ (https://swissmodel.expasy.org/), a widely utilized homology modeling platform, was employed to construct three-dimensional structural models of the nanobodies Nb60, Nb65, and Nb108. Suitable templates were selected from the Protein Data Bank (PDB) based on high sequence identity: 7WD1 ^41^ (79.23%) for Nb60, 5IML^42^ (80.80%) for Nb65, and 8EMZ^43^ (86.89%) for Nb108. Target sequences were submitted to the SWISS-MODEL server, which automatically performed alignment, model building, and internal geometry optimization.

Model quality was assessed using SWISS-MODEL–provided metrics: Nb60 (GMQE = 0.76, QMEAN = 0.76 ± 0.07), Nb65 (GMQE = 0.80, QMEAN = 0.79 ± 0.07), and Nb108 (GMQE = 0.83, QMEAN = 0.82 ± 0.08). Ramachandran plot analysis was performed by MolProbity server^44^ (http://molprobity.biochem.duke.edu/). The results revealed that 99.24%, 95.16%, and 95.83% of residues, respectively, resided in favored regions, with only Nb65 and Nb108 showing minor outliers (0.83% and 0.81%, none in CDR3). Local quality estimates indicated that CDR3 loops (residues 104–112 in Nb60, etc.) displayed moderately reduced confidence (local QMEAN Z-scores ranging from −1.3 to −0.7), which is typical for hypervariable regions. Nonetheless, CDR3 backbone conformations remained stereochemically reasonable and devoid of Ramachandran outliers. Structural superposition of the framework regions (excluding CDR loops) with the respective templates yielded low Cα RMSD values (0.109 Å, 0.149 Å, and 0.170 Å), confirming high structural conservation of the core scaffold. These refined homology models were subsequently used in downstream docking and interaction analyses, providing reliable structural insights into antigen–nanobody recognition.

### Antigen–Antibody Docking Using ClusPro and Rosetta

Initial docking was performed using ClusPro^45^ server (https://cluspro.bu.edu/), with the Trop-2 extracellular domain (PDB: 7E5M^46^) as the receptor and SWISS-MODEL-generated nanobody structures as ligands. ClusPro returned models clustered by interface geometry and ranked by energy-based scoring. Rather than selecting the top-scoring pose alone, we prioritized biological relevance: each of the top five ClusPro clusters was visually inspected for overlap with the experimentally defined sacituzumab epitope on Trop-2 (residues Q237–Q252, as resolved in PDB 7E5M^46^). Among clusters exhibiting direct contact with this functional epitope, we selected the representative model with the lowest ClusPro energy score as the starting structure for high-resolution refinement.

This model was refined using RosettaDock^47^ two-stage protocol (centroid-mode sampling followed by all-atom optimization with side-chain repacking). We generated 1,000 decoys and selected the final pose based on the lowest Rosetta interface energy. To assess convergence and reliability, we evaluated the top-scoring models using the CAPRI criteria: the N5 value (number of acceptable-or-better models among the top 5) was ≥3, satisfying the threshold for a “high-quality” prediction. All complexes satisfied the CAPRI evaluation.^48^ Subsequently, the selected docking complexes were further analyzed using Rosetta’s InterfaceAnalyzer to quantify interfacial properties, including buried solvent-accessible surface area (ΔSASA).

### Molecular Dynamics (MD) Simulation and Molecular Mechanics/Generalized Born Surface Area (MM/GBSA) Calculation

Three complexes of Trop-2 (232 residues) coupled with different nanobodies: Nb60 (133 residues), Nb65 (127 residues), and Nb108 (123 residues), and two distinct binding poses (binding pose 1 and binding pose 2) were used for the MD simulations. The Trop-2 structure was taken from the soluble ectodomain construct (PDB 7E5M^46^), which excludes the transmembrane and cytoplasmic regions, consistent with the experimental nanobody-binding assays. The membrane environment was therefore not included in the simulations. Each system was solvated in a cubic TIP3P water box with a side length of 110 Å, containing approximately 36,000 water molecules and sufficient Na^+^/Cl^–^ ions to achieve a 0.15 M NaCl concentration and overall charge neutrality. The resulting solvated systems comprised approximately 115,000 atoms each. The same force fields or parameters^49–51^ described in our previous publications^52–55^ were applied to the Trop-2 receptor (ff14SB force field), water molecules, and nanobodies.

Each MD system was first relaxed by five 10,000-step minimizations followed by five restrained MD simulations to remove possible steric clashes. Each restrained MD simulation lasted 1 ns (ns) using an integration time step of 1 fs (fs). The five minimization and restrained MD runs applied 20, 10, 5, 1, and 0 kcal/mol to the mainchain atoms, sequentially. There were three phases for the subsequent NPT (constant particle number, pressure, and temperature) MD simulations: the relaxation phase (2 ns for each temperature from 50 to 250 K at a step of 50 K), the equilibrium phase (12 ns, 298 K), and the sampling phase (1000 ns, three independent replicates). The integration of the equations of motion was conducted at a time step of 2 fs for all the three phases. Electrostatic interactions between ions and other charged particles were calculated using Coulomb’s law. Long-range Coulomb interactions were handled using the Particle-Mesh Ewald (PME) method with a 10 Å cutoff. van der Waals interactions were computed using atom-based nonbonded lists with a 10 Å cutoff, and continuous corrections were applied to account for long-range effects. The constant pressure simulations were carried out at 1 atm via the Berendsen barostat with the pressure relaxation time τ_p_ of 3.0 ps. The Berendsen barostat was chosen for its efficiency and effectiveness in quickly stabilizing pressure during the initial equilibration phase. Its simplicity and robustness provided a stable starting point for our simulations. The temperature was regulated using Langevin dynamics with a collision frequency of 1 ps^–1^. The SHAKE algorithm was applied to all bonds involving hydrogen atoms. The periodic boundary condition was applied to all MD simulations which were performed using the pmemd. cuda module implemented in the AMBER18 ^56–58^ software package. For each system involving the complexation of Trop-2 and its corresponding nanobody, three separate and independent replicates were executed, employing distinct random seeds. To achieve this, we configured the software by setting the “ig” parameter to “–1” in the configuration file.

100 snapshots were selected from the sampling phase for MM-GBSA binding free energy decomposition analysis. For each MD snapshot, the molecular mechanical (MM) energy (*E*_MM_) and the Poisson–Boltzmann Surface Area (PBSA) energy terms were calculated without further minimization.^59,60^ The interaction energies between each residue and nanobody were calculated with the solvent effect being considered using a MM/GBSA solvation model.^61^

### Expression and Purification of Designed Nanobody

Plasmid constructs encoding the recombinant nanobodies were transformed into *E. coli* BL21 (DE3) competent cells (CB105-02, TIANGEN). A single colony was inoculated into lysogeny broth (LB) medium and cultured at 37 °C with shaking at 220 rpm until the optical density at 600 nm (OD_600_) reached 0.6–0.8. Protein expression was induced by adding 0.8 mM isopropyl β-d-1-thiogalactopyranoside (IPTG), followed by incubation at 16 °C for 18 h.

After induction, bacterial cells were harvested by centrifugation at 4,000 × *g* for 15 min at 4 °C. The pellet was resuspended in lysis buffer containing 50 mM Tris-HCl (pH 8.0), 300 mM NaCl, 10 mM imidazole, and 1 mM PMSF. The resuspended cells were lysed by ultrasonication on ice, and the lysates were clarified by centrifugation at 15,000 × *g* for 30 min at 4 °C.

The supernatant containing the soluble His-tagged nanobody was applied to Ni-NTA agarose beads (SA004025, Smart-Lifesciences) for affinity purification. After binding, the resin was washed with wash buffer (50 mM Tris-HCl, pH 8.0, 300 mM NaCl, 20 mM imidazole) to remove nonspecifically bound proteins. The target protein was then eluted with elution buffer (50 mM Tris-HCl, pH 8.0, 300 mM NaCl, 250 mM imidazole).

Eluted fractions were analyzed by SDS-PAGE followed by Coomassie Brilliant Blue staining to assess protein purity. Protein concentration was determined using the BCA protein assay.

### Bio-Layer Interferometry (BLI)

All assays were carried out in a 96-well microplate, with a final volume of 200 μL per well. All the binding assays were performed at 30 °C. For all experiments, streptavidin (SA) biosensors (18–5020, Octet) were used for the immobilization of Trop-2. The SA biosensors were hydrated in PBST (0.02% Tween 20) for at least 10 min before use. Before all measurements, a baseline step of 60 s was performed in PBST. Next, Trop-2 was loaded onto the biosensors at a concentration of 100 μg/mL for 120 s. For binding kinetics experiments, probes were dipped in different nanobody PBST solutions of the same concentration. The baseline, association, and dissociation steps were 60, 120, and 180 s, respectively. The binding kinetics curves of different nanobodies at the same concentrations were aligned to 60 s of the baseline step and fitted to a 1:1 binding kinetics model using the Octet Analysis Studio 12 software.

## Results and Discussion

### 3D Overview of Trop-2 and Nanobodies

The entire crystal structure of the Trop-2 protein and the full-length modeled structures of the nanobodies were used to conduct the molecular docking and MD simulations. The reported binding sites of these nanobodies on Trop-2 are expected to be situated on a stretched polypeptide within the C-terminal cysteine-poor domain (CPD, Q237–Q252).^46^ As mentioned earlier, the two most frequent binding poses observed for all three nanobodies are shown in Figure 1. To quantitatively evaluate the binding interfaces, we further analyzed the Trop-2–nanobody complexes using the Rosetta InterfaceAnalyzer. The calculated buried solvent-accessible surface areas (ΔSASA) ranged from approximately 1395 to 1508 Å^2^ across the six docking models, indicating that all three nanobodies form extensive and comparable contact interfaces with Trop-2. Specifically, the ΔSASA values were 1508 Å^2^ and 1441 Å^2^ for Nb60 (poses 1 and 2), 1502 Å^2^ and 1399 Å^2^ for Nb65 (poses 1 and 2), and 1477 Å^2^ and 1395 Å^2^ for Nb108 (poses 1 and 2). The similarity of interface areas among these complexes supports the notion that the three nanobodies recognize a conserved surface region on the Trop-2 CPD domain. The structural superposition of all six complexes (Figure S1) further confirmed that the binding orientations of the nanobodies are highly similar, consistent with their comparable ΔSASA values and binding energetics. Based on the mean total binding free energy and docking scores, binding pose 1 in all three systems emerges as the most promising binding mode between Trop-2 and its nanobodies.

**Figure 1:**
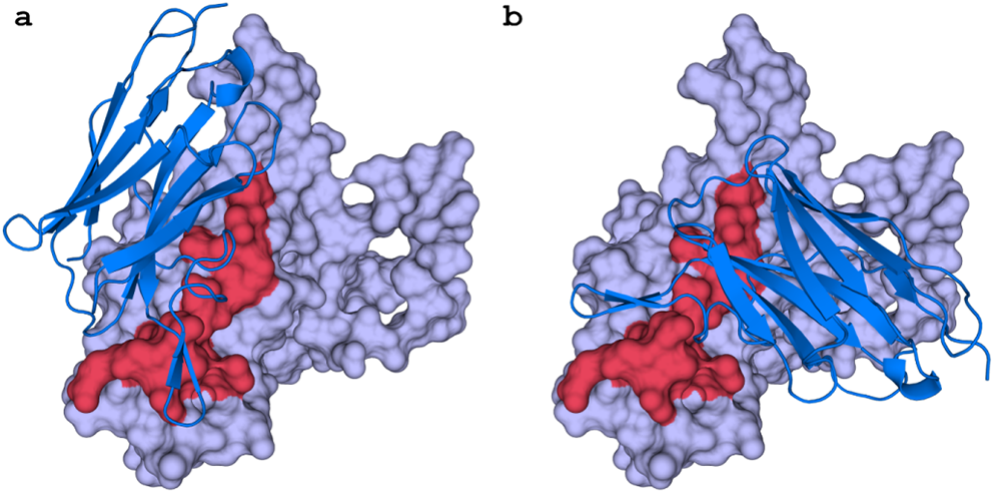
Two representative binding poses between Trop-2 and Nb60. (a) The predicted binding pose 1 of three nanobodies. (b) The predicted binding pose 2 of three nanobodies. We observed that all three investigated nanobodies shared the similar binding poses. The binding sites of these nanobodies on Trop-2 are anticipated to be located along a stretched polypeptide within the C-terminal cysteine-poor domain (CPD, Q237–Q252 as indicated by red surface).

Table 1 presents a comparative analysis of the mean total binding free energy, calculated (averaged over three independent replicates) using the Generalized Born solvent model. The comparison is made across different binding poses for Nb60, Nb65, and Nb108 nanobodies. Notably, we observed that the average total binding free energy associated with Binding Pose 1 was consistently lower than that of Binding Pose 2 for all three nanobodies. This trend can be quantitatively seen in the provided data: Nb60 exhibited −129.80 kcal/mol (Pose 1) and −93.78 kcal/mol (Pose 2), Nb65 had −99.48 kcal/mol (Pose 1) and −80.97 kcal/mol (Pose 2), while Nb108 showed −88.51 kcal/mol (Pose 1) and −66.89 kcal/mol (Pose 2). Furthermore, the table also includes experimental equilibrium dissociation constants (*K*_d_) which provide additional insight into the binding affinity; these range from 0.7 nM for Nb60 to 118.8 nM for Nb108, with Nb65 having a *K*_d_ of 35.4 nM, indicating varying degrees of interaction strength between Trop-2 and the nanobodies.

**Table 1:** Mean Total Binding Free Energies (Δ*G*, kcal/mol) ± Standard Deviations, Obtained from Three Independent MM/GBSA Replicates Using the Generalized Born Solvent Model, for the Interactions between Trop-2 and the Three Nanobodies^a^

Nanobody	Binding Pose 1 (Δ*G*, kcal/mol)	Binding Pose 2 (Δ*G*, kcal/mol)	Equilibrium Dissociation Constant (*K*_d_, nM)
Nb60	–129.80 ± 6.82	–93.78 ± 6.86	0.7
Nb65	–99.48 ± 3.10	–80.97 ± 4.13	35.4
Nb108	–88.51 ± 7.27	–66.89 ± 5.42	118.8

a Experimental equilibrium dissociation constants (*K*_d_) for Nb60, Nb65, and Nb108 were taken from previously published BLI measurements.^18^

The simulations were based on the soluble ectodomain of Trop-2, excluding transmembrane and cytoplasmic regions. Although membrane effects cannot be fully ruled out, this ectodomain model aligns with existing nanobody-binding data. Future work will examine full-length or membrane-embedded Trop-2 to assess membrane influences on nanobody recognition.

### Key Interactions and Residues in Trop-2 That Interact with Nb60 from MD

Figure 2a and b depict the RMSD of two binding poses for the Trop-2 and Nb60 complex. Throughout the simulation, the RMSDs of Nb60 and Trop-2 in binding pose 1 exhibited stability, hovering around 2.5 Å and 3.5 Å respectively, from 20 to 1000 ns. Conversely, in binding pose 2, the RMSD of Nb60 increased to 4.0 Å, while that of Trop-2 remained consistently around 1.8 Å. These findings strongly suggested that binding pose 1 was notably more stable than binding pose 2 within the Trop-2 and Nb60 system, which is consistent with our prior docking predictions.

**Figure 2:**
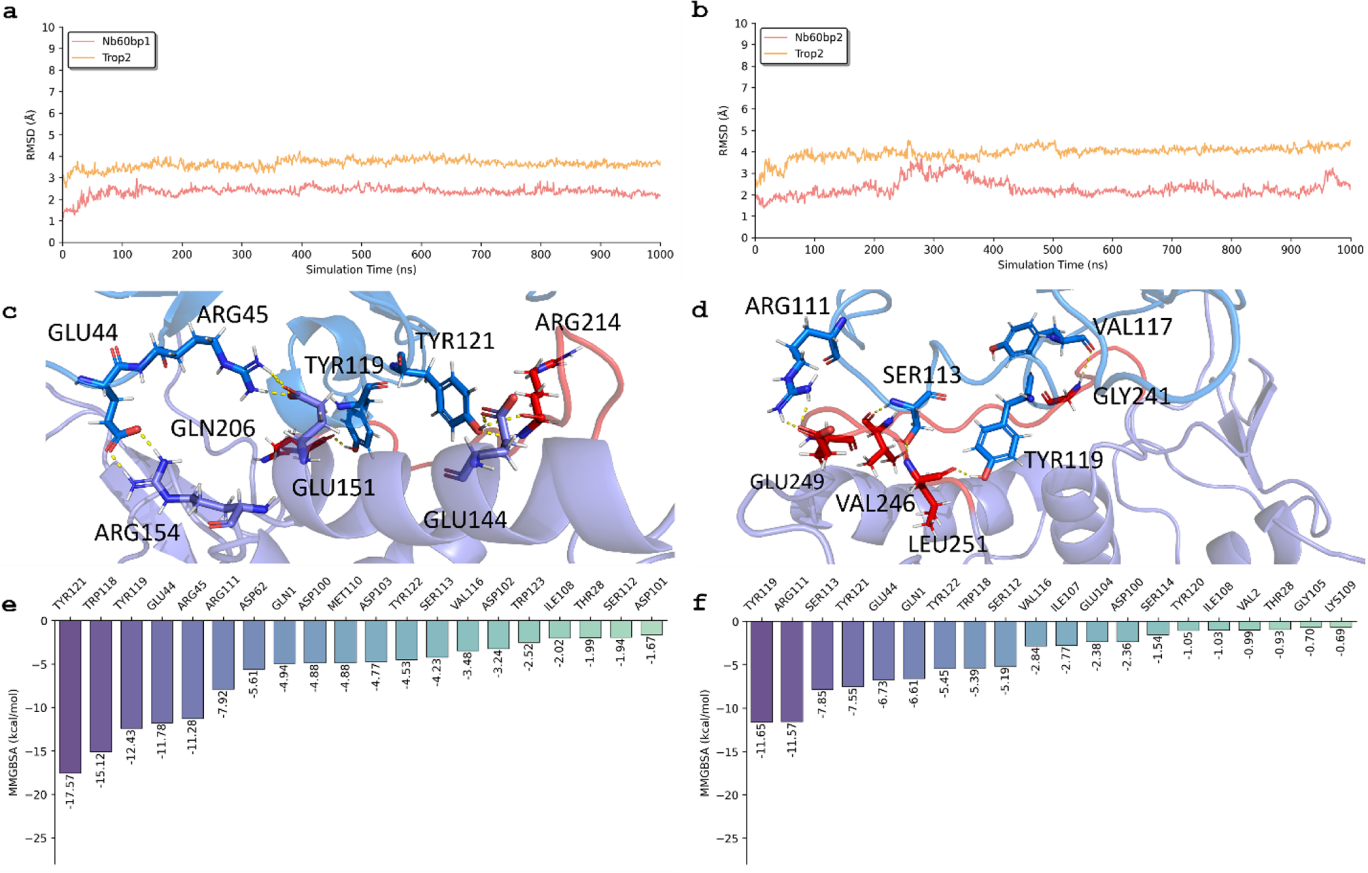
Molecular dynamics (MD) results for Trop-2 and Nb60 in terms of binding poses 1 and 2. The RMSD of Trop-2 and Nb60 in binding pose 1 (a) and 2 (b). The interactions between Trop-2 and Nb60 in binding pose 1 (c) and 2 (d). The free energy decomposition of residues in Trop-2 in binding pose 1 (e) and 2 (f). The backbone atoms (N, Cα, C, and O) are used to calculate RMSD and perform alignment.

Figure 2c illustrates the interactions between Trop-2 and Nb60 after molecular dynamics (MD) simulations for binding pose 1. Our observations revealed persistent and strong interactions between several residues of Trop-2 and Nb60, including TRP118, GLU44, ARG45, ASP102, TYR119, GLU104, ASP100, TYR122, ASP62, ARG111, MET110. Notably, GLU144, GLU151, GLN206, and ARG154, involved in hydrophilic interactions, exhibited robust binding with Nb60. Additionally, other specific residues interacted strongly with Nb60 through hydrophobic interactions, including TYR121, TYR119, TYR122, and MET110. All of the residues mentioned above are from Trop-2 receptor.

Figure 2d provides detailed insights into the interactions of Trop-2 and Nb60 before and after MD for binding pose 2. In this pose, Trop-2 residues established hydrogen-bonding interactions with Nb60, including specific residues (GLY241, VAL246, GLU249, and LEU251) identified during the simulation. Furthermore, other specific residues that included Glu104, SER113, TYR121, SER112, VAL116, and ILE107 were identified to have interacted strongly with Nb60 through hydrophobic interactions. All of the residues mentioned above are from Trop-2 receptor.

Finally, Figure 2e and f present residue energy decomposition for these binding poses, offering a quantitative breakdown of the contributions made by each residue to the stability and binding affinity of the Trop-2 and Nb60 complex.

Two additional independent replicates were performed for each system, and the corresponding data can be found in Figure S2 within the Supporting Information. Our findings revealed that the RMSDs and overlapped conformations of binding pose 1 for Trop-2 complexed with Nb60 demonstrated greater stability than those observed for binding pose 2. Furthermore, the residues involved in the protein–protein interface were conserved for all the three replicates. All these results suggest that binding pose 1 is energetically more favorable compared to binding pose 2.

### Key Interactions and Residues in Trop-2 That Interact with Nb65 from MD

Figure 3a and b present the RMSD of two binding poses for the Trop-2 and Nb65 complexes. During the simulation, the RMSDs of Nb65 and Trop-2 in binding pose 1 remained stable, hovering around 6.0 Å and 1.3 Å, respectively, from 20 to 1000 ns. In binding pose 2, the RMSD of Nb65 fluctuated around 4.1 Å, while that of Trop-2 remained consistently around 1.6 Å. These results suggest that both binding poses were stable during the MD simulation. However, the total binding free energy of binding pose 1 (−100.06 kcal/mol) was more favorable than that of binding pose 2 (−76.34 kcal/mol).

**Figure 3:**
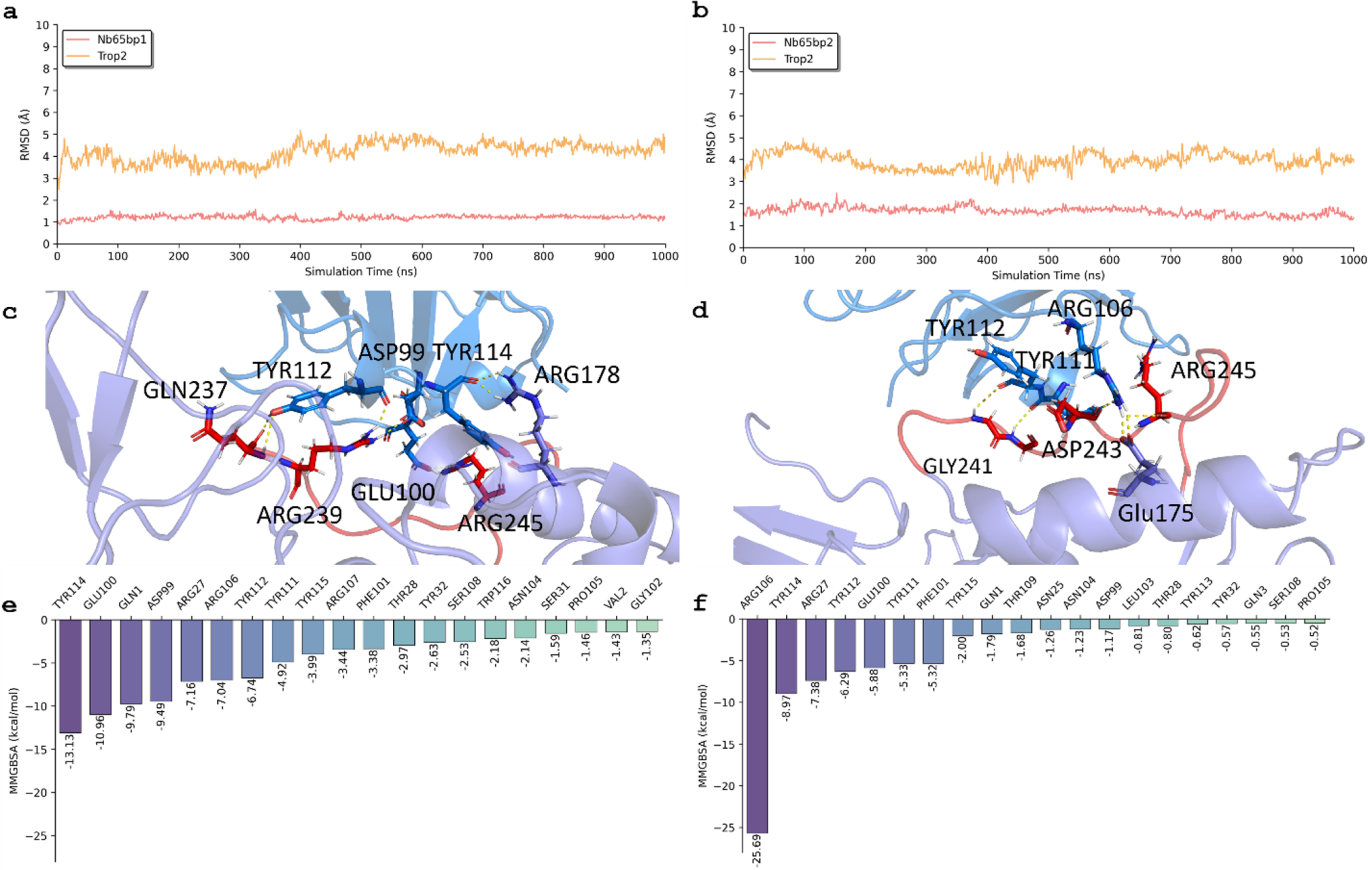
Molecular dynamics (MD) results for Trop-2 and Nb65 in terms of binding poses 1 and 2. The root-mean-square deviations (RMSDs) of Trop-2 and Nb65 in binding pose 1 (a) and 2 (b). The interactions between Trop-2 and Nb65 in binding pose 1 (c) and 2 (d). The free energy decomposition of residues in Trop-2 in binding pose 1 (e) and 2 (f). The backbone atoms (N, Cα, C, and O) are used to calculate RMSD and perform alignment.

Figure 3c illustrates the interactions between Trop-2 and Nb65 after MD simulations for binding pose 1. Our observations revealed persistent and strong interactions between several residues of Trop-2 and Nb65, including TYR114, ARG27, TYR115, ASP99, TYR112, GLN3, GLN1, ARG106, TYR32, TYR111, and more. Notably, residues involved in hydrophilic interactions exhibited robust binding with Nb65, including ASP99, TYR112, TYR114, and TYR115. Additionally, other specific residues, such as ARG178, ARG245, ARG239, and GLN237, interacted strongly with Nb65 through hydrophobic interactions. All of the residues mentioned above are from Trop-2 receptor.

Figure 3d describes detailed insights into the interactions of Trop-2 and Nb65 after MD for binding pose 2. Trop-2 residues established hydrogen-bonding interactions with Nb65 in this pose, including specific residues (GLY241, ASP243, ARG245) identified during the simulation. Furthermore, other specific residues, like TRP110, GLN3, THR109, TYR112, and TRP116, were identified to have interacted strongly with Nb65 through hydrophobic interactions. All of the residues mentioned above are from Trop-2 receptor.

Figure 3e and f provide residue energy decomposition for these binding poses, offering a quantitative breakdown of the contributions made by each residue to the stability and binding affinity of the Trop-2 and Nb65 complex.

For each system, two other separate trials were executed, and the outcome of these experiments can be visually inspected in Figure S3. The results exhibited that when Trop-2 was complexed with Nb65, binding pose 1 illustrated a higher degree of stability as evident from both lower RMSDs and more consistent overlapped conformations compared to binding pose 2.

### Key Interactions and Residues in Trop-2 That Interact with Nb108 from MD

Figure 4a and b reveal the RMSD of two binding poses for the Trop-2 and Nb108 complex. Throughout the simulation, the RMSDs of Nb108 and Trop-2 in binding pose 1 exhibited stability, hovering around 4.6 Å and 2.1 Å respectively, from 100 to 1000 ns. In binding pose 2, the RMSD of Nb108 fluctuated around 4.5 Å, while that of Trop-2 remained consistently around 2.0 Å. These results suggest that both binding poses were stable during the MD simulation. However, the total binding free energy of binding pose 1 (−84.12 kcal/mol) was more favorable than that of binding pose 2 (−73.14 kcal/mol).

**Figure 4:**
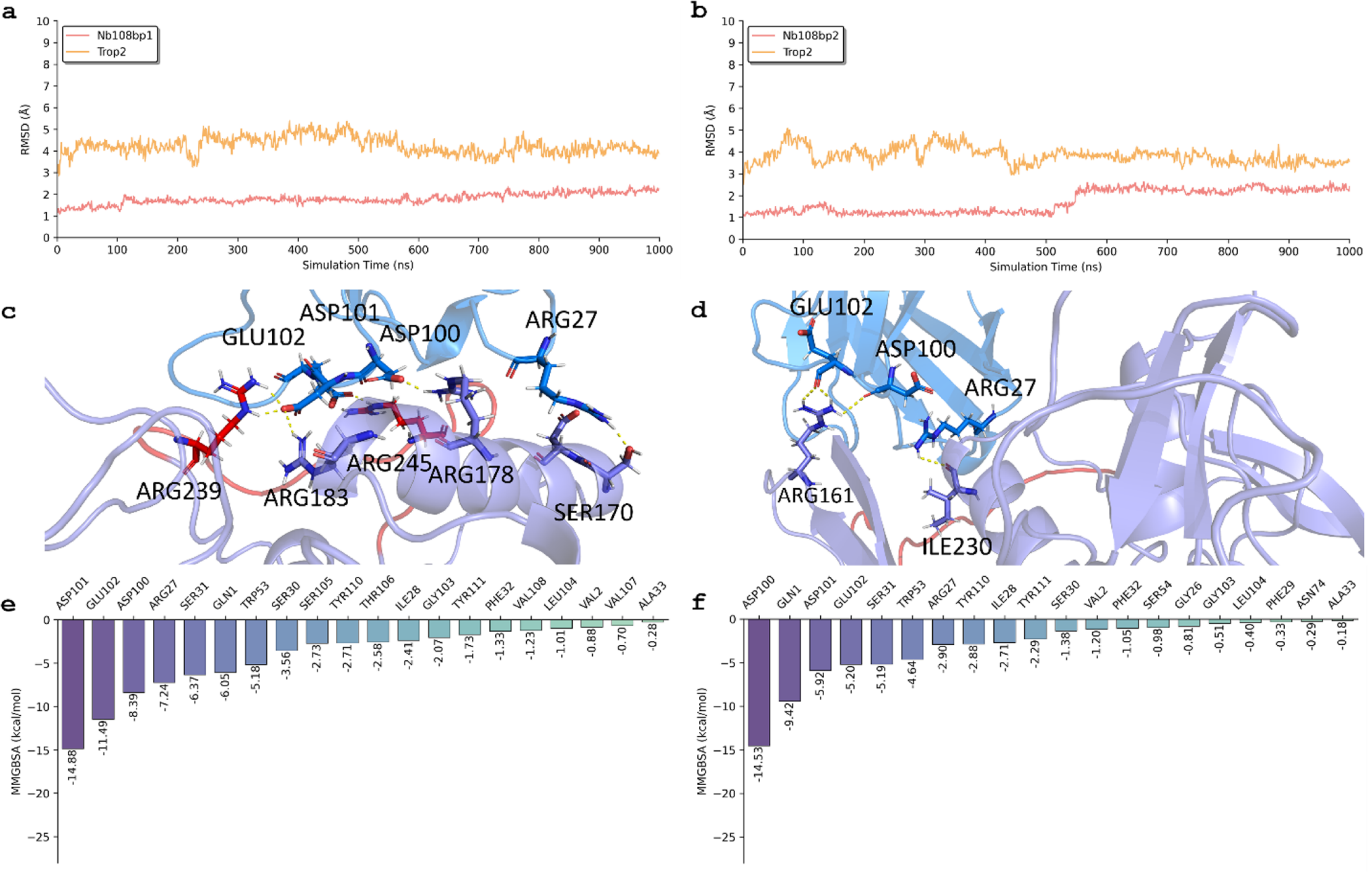
Molecular dynamics (MD) results for Trop-2 and Nb108 in terms of binding poses 1 and 2. The root-mean-square deviations (RMSDs) of Trop-2 and Nb108 in binding pose 1 (a) and 2 (b). The interactions between Trop-2 and Nb108 in binding pose 1 (c) and 2 (d). The free energy decomposition of residues in Trop-2 in binding pose 1 (e) and 2 (f). The backbone atoms (N, Cα, C, and O) are used to calculate RMSD and perform alignment.

Figure 4c shows the interactions between Trop-2 and Nb108 after MD simulations for binding pose 1. Our observations revealed persistent and strong interactions between several residues of Trop-2 and Nb108, including ASP101, ASP100, GLN1, SER31, ARG27, GLU102, TRP53, PHE32, LEU104, TYR59, and VAL108. Notably, residues that included ARG239, ARG183, ARG245, and ARG178 were involved in hydrophilic interactions and exhibited robust binding with Nb108. Additionally, other specific residues, such as TRP53, PHE32, LEU104, TYR59, and VAL108, interacted strongly with Nb108 through hydrophobic interactions. All of the residues mentioned above are from Trop-2 receptor.

Figure 4d provides detailed insights into the interactions of Trop-2 and Nb108 after MD for binding pose 2. In this pose, Trop-2 residues established hydrogen-bonding interactions with Nb108, including specific residues (ARG161 and ILE230) identified during the simulation. Furthermore, other specific residues, like TRP53 GLY56, SER105, TYR59, and THR58, were identified to have interacted strongly with Nb108 through hydrophobic interactions. All of the residues mentioned above are from Trop-2 receptor.

Figure 4e and f reveal residue energy decompositions for these binding poses, offering a quantitative breakdown of the contributions made by each residue to the stability and binding affinity of the Trop-2 and Nb108 complex.

Two additional MD runs were carried out for each conformation of Trop-2 and Nb108, with the outcomes depicted in Figure S4. The findings indicated that upon complexation between Trop-2 and Nb108, binding pose 1 demonstrated a superior stability level, as demonstrated by lower RMSDs and more uniform overlapped conformations compared to binding pose 2.

### Experimental Validations for the Predictions from MD Simulations

To quantitatively assess the importance of the identified interfacial residues, residue-level contact frequencies and hydrogen-bond occupancies were evaluated over the equilibrated portions of the MD trajectories. Contacts were defined using a heavy-atom distance cutoff of 4.0 Å, and hydrogen bonds were identified based on standard geometric criteria. For each nanobody–Trop-2 complex, the residues listed in Table 2 exhibited sustained interactions with the antigen, with contact occupancies typically exceeding 30–50% of the simulation time and, for several hotspot residues, persisting for more than 60% of the trajectory.

**Table 2:** Critical Residues within the Nanobodies Were Identified through Molecular Dynamics Simulations^a^

Nanobody	Key Resides in the nanobody
Nb60	TRP118, GLU44, TYR121, ARG45, ASP102, TYR119, GLU104, ASP100, TYR122, ASP62
Nb65	TYR114, ARG27, TYR115, ASP99, TYR112, GLN3, GLN1, ARG106, TYR32, TYR111
Nb108	ASP101, ASP100, GLN1, SER31, ARG27, GLU102, TRP53, PHE32, LEU104, TYR59

a Residues were selected based on their high contact persistence, defined as repeated participation in interfacial contacts over a substantial fraction of the MD trajectory, rather than isolated interactions observed in single docking pose.

Interface stability was further assessed by monitoring RMSD of the interfacial residues and the number of interfacial contacts over time, which remained stable after equilibration. Importantly, the same set of key residues was consistently observed across independent simulation replicas, indicating that their contributions to binding were robust rather than artifacts of a single trajectory.

Table 2 presents the amino acid combinations that from different nanobodies significantly contributed to binding, derived from docking and molecular dynamics simulations. The identification of key residues and the superior binding pose has several implications for nanobody design and therapeutic applications. The findings that Nb60, with a longer CDR3, exhibits stronger binding to Trop-2 align with previous studies that have highlighted the importance of CDR3 length in antibody–antigen interactions. For example, studies on other antigens have shown that a longer CDR3 can increase the binding surface area, thereby enhancing the strength and specificity of interactions. These insights could be pivotal in guiding the design of more effective nanobodies with improved binding affinities by optimizing CDR3 length and composition.

The identified key residues, such as TRP118 in Nb60 and TYR114 in Nb65, are consistent with known binding hot spots in antibody–antigen interactions. These residues could serve as focal points in the design of next-generation nanobodies, where modifications or enhancements could further increase binding strength and specificity. For instance, targeted mutagenesis studies could explore how altering these residues impacts binding, offering a pathway for fine-tuning nanobody interactions with Trop-2.

Our findings are consistent with previous structural studies on Trop-2, which identified similar regions as critical for binding. However, our work extends these findings by providing a more detailed map of the binding interactions at the molecular level, specifically focusing on nanobodies. The comparison of binding free energies and the emphasis on the superiority of binding pose 1 adds a novel dimension to the understanding of Trop-2–nanobody interactions, which was not fully explored in earlier studies.

To further investigate the role of CDR3 in Trop-2-specific nanobodies, we recently applied a novel algorithm AntiBMPNN, developed in-house, to redesign the CDR3 sequence in Nb60. Figure 5a shows the sequence alignment between Nb60 and three redesigned nanobodies. Modifying the sequence of “EGGIIKMRSSSTVVW” (residues 104 to 118) to “YGGRSLYNGPGTSPG” (Nb-7) or “YGGYYYYNGPGTSPE” (Nb-6) resulted in significant changes in binding affinity. More extensive modifications, such as “PALYYGGYSYYNGPGTSPE” (residues 100 to 118) for Nb-14, appear to worsen the binding characteristics. In Figure 5b, the BLI sensorgram illustrates the interaction between Trop-2 and three distinct nanobodies (Nb-6, Nb-7, and Nb-14). During the association phase, which occurs from 60 to 180 s, the initial contact and binding of these nanobodies to Trop-2 is observed. Notably, Nb-7 displays the highest binding response, reaching approximately 0.4. Following the association phase, starting at 180 s until the end of the experiment, Nb-7 undergoes a slow dissociation process, as indicated by a gradual decline in its curve. This suggests that Nb-7 binds stably to the target and has a slow dissociation rate. The affinity constant (*K*_d_) for Nb-7 was determined to be 7.931 μM. In comparison, Nb-6 and Nb-14 show lower binding affinities. Both Nb-6 and Nb-14 have lower binding responses and dissociate more quickly than Nb-7, further supporting their lower affinity for the target. Due to the weaker binding and dissociation properties of Nb-6, and Nb-14 compared to Nb-7, reliable affinity data could not be calculated for these two nanobodies. Figure 5c illustrates the detailed interactions between Nb-7 and Trop-2, indicating the role of CDR3 in Nb60. The substantial reduction in binding affinity for the redesigned Nb-7 (from 0.7443 nM to 7.931 μM or worse) indicates that the CDR3 loop is critical for the binding affinity of Trop-2 nanobodies, underscoring its importance in the design and efficacy of Trop-2-targeting nanobodies.

**Figure 5:**
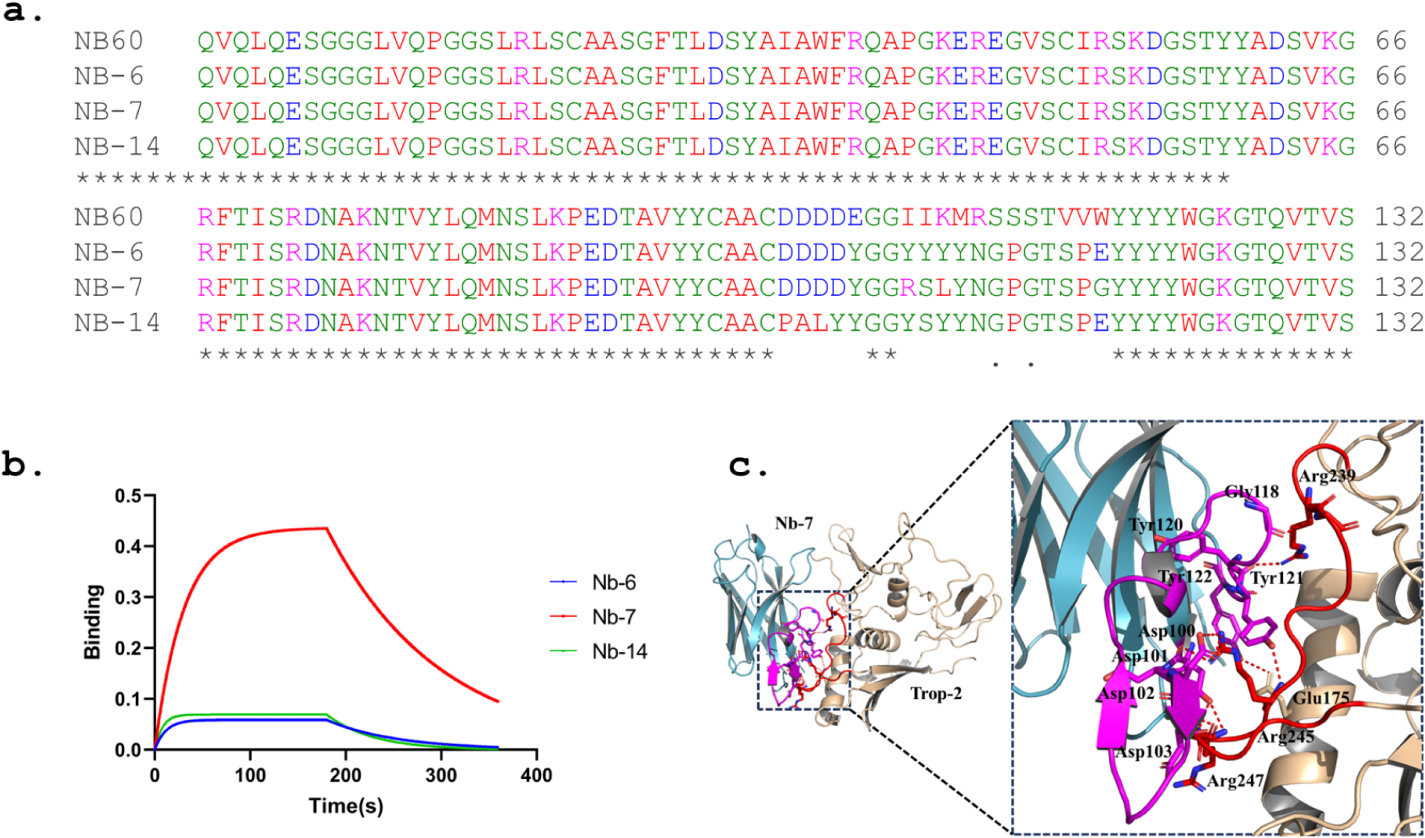
The designed nanobody Nb-7 (*K*_d_: 7.931 μM). (a) The sequence alignment between Nb60 and the other three CDR3 redesigned nanobodies Nb-6, Nb-7, and Nb-14. (b) Representative sensorgram for three designed nanobodies, Nb-6 (blue), Nb-7 (red), and Nb-14 (green). (c) The detailed interaction between CDR3 of Nb-7 and Trop-2.

Direct experimental characterization of Nb60, Nb65, and Nb108 was not performed in this study, as the experimental effort was focused on probing the sensitivity of the MD-identified hotspot region through targeted redesign of Nb60. This strategy allowed us to assess whether the computationally identified binding determinants represent an optimized interaction network that is difficult to improve upon without loss of affinity. The substantial loss of binding affinity observed in the redesigned nanobodies does not indicate a failure of the MD predictions. Instead, it highlights the highly optimized nature of the native CDR3–Trop-2 interaction identified by simulation. MD analysis suggests that the original CDR3 sequence forms a delicately balanced interaction network, in which both side-chain chemistry and loop conformation are tightly coupled. Disruption of this balance through extensive sequence modification can therefore result in a pronounced reduction in binding affinity, even when redesign is performed using advanced, structure-aware algorithms.

The present simulations were based on the soluble ectodomain of Trop-2 (PDB 7E5M), which excludes the transmembrane and cytoplasmic regions. While the possible influence of the membrane cannot be completely ruled out, this ectodomain-based model is consistent with available experimental nanobody-binding data. In future work, we plan to explore full-length Trop-2 models or membrane-embedded systems to further evaluate the potential impact of the membrane environment on nanobody recognition.

## Conclusions

Our study delved deeper into the molecular interactions between Trop-2 and three distinct nanobodies (Nb60, Nb65, and Nb108) using MD simulations. Through these simulations, we have gained valuable insights into the dynamic behavior and binding details that govern these intricate interactions.

In our analysis of the Trop-2–Nb60 complex, specific residues such as GLU44, ARG45, ASP102, and TRP118 (CDR3), were identified as key contributors to hydrogen bond interactions, underscoring their importance in stabilizing the complex. Similarly, for the Trop-2–Nb65 complex, residues like ASP99, TYR112, TYR114, TYR115 (CDR3), and TYR32 (CDR1) were implicated in crucial interactions essential for binding affinity. In the case of the Trop-2–Nb108 complex, residues including ARG27, SER31 (CDR1), ASP100, and ASP101 (CDR3) were identified as significant contributors to the binding interface, highlighting the diverse roles played by different nanobodies in recognizing and interacting with Trop-2.

Across the three Trop-2–nanobody complexes, the binding interface is built on two structural motifs of Trop-2: CPD (GLN237–GLN252) and the adjacent α-helix (SER170–TYR185). In every complex, these two motifs contribute polar contacts (hydrogen bonds and salt-bridge interactions) that anchor the nanobodies. In contrast, each nanobody engages a distinct set of hotspot residues through its CDR. Nb60 used an aromatic triad in CDR3 (TRP118–TYR119–TYR122) together with the acidic pair GLU44/GLU104, forming a hydrophobic-polar pocket. Nb65 relies on a TYR-rich patch (TYR114, TYR115, TYR112) and ARG27 in CDR1, establishing a stacked π–π/π–cation network. Nb108 is dominated by ARG27/SER31 (CDR1) and ASP100/ASP101 (CDR3), generating a mixed charge-hydrophobic surface. Thus, the conserved CPD-helix core supplies a common scaffold for polar anchoring, while nanobody-specific CDR motifs dictate the fine-tuned affinity and specificity of each interaction.

Based on the MD’s results, we then redesigned the CDR3 region of Nb60 to create three novel nanobodies: Nb-6, Nb-7, and Nb-14. Experimental data indicated that Nb-7 demonstrated the highest affinity for Trop-2 with a *K*_d_ value of 7.931 μM, while Nb-6 and Nb-14 had reduced or negligible binding affinities. This highlights the critical role of the CDR3 loop in binding affinity and underscores its importance in nanobody design. The variations in binding affinities among the nanobodies provide valuable insights into Trop-2–nanobody interactions and guide the development of more effective Trop-2-targeting therapies. These findings provide insights into the nuanced molecular recognition mechanisms governing Trop-2–nanobody interactions, emphasizing the importance of specific residues within the complementarity-determining regions (CDRs) for binding affinity and stability. The dynamic nature revealed by MD simulations complements our understanding of these interactions, offering a foundation for future engineering and optimization of nanobodies for therapeutic applications targeting Trop-2-associated diseases.

## Supplementary Material



## Data Availability

The raw data and example files can be downloaded from GitHub (https://github.com/zeysun/Trop2-Nanobody) or are available from the corresponding author upon request.

## Notes

Figure S1 presents the structural alignment of the Trop-2–nanobody complexes; Figure S2 presents supporting information on the root-mean-square deviations (RMSDs) and superimposed conformations for the complex between Trop-2 and Nb60; Figure S3 illustrates the RMSDs and overlapped conformations for the two additional replicates of the Trop-2–Nb65 complex system; Figure S4, on the other hand, depicts the RMSDs and conformations for the system where Trop-2 is in a complex with Nb108 (PDF)

## ○

Present address: Faculty of Pharmaceutical Sciences, Shenzhen University of Advanced Technology, Shenzhen 518107, China
