# Supplementary material for: Exploring Trop-2–Nanobody PPI Interactions through Molecular Dynamics Simulations and Biovalidations
Source: ACS Omega. 2026 Mar 5;11(10):16209–19. doi: 10.1021/acsomega.5c11494 (PMC13000607; doi:10.1021/acsomega.5c11494)
Supplement: Supplementary file 1 [file ao5c11494_si_001.pdf]

Exploring Trop2-Nanobody PPI Interactions through Molecular Dynamics Simulation and Bio-validations

Jin Cheng[1#], Ze-Yu Sun[2#], Zhiyuan Guo[3#], Yixuan Hao[2], Gavin Hou[4], Yijin Li[1], Yuanqiang Wang[5*], Zhiwei Feng[2,6*†], Ying Xue[6,7*], Li Meng[1*]

[1]School of Pharmacy, Jiangsu Vocational college of Medicine, Yancheng, P.R. China, 224005

[2]Department of Pharmaceutical Sciences, Computational Chemical Genomics Screening Center, and Pharmacometrics & System Pharmacology PharmacoAnalytics, School of Pharmacy; National Center of Excellence for Computational Drug Abuse Research, University of Pittsburgh, Pittsburgh, Pennsylvania 15261, United States

[3]Yancheng No.1 People's Hospital, Yancheng, P.R. China, 224006

[4]College of Engineering, University of Michigan Ann Arbor, Ann Arbor, MI 48109, United States

[5]School of Pharmacy and Bioengineering, Chongqing University of Technology, Chongqing, P.R. China, 400054

[6]Fuculty of Pharmaceutical Sciences, Shenzhen University of Advanced Technology, Shenzhen 518107, China

[7]Department of Pharmacy, Zhongshan Hospital, Fudan University, Shanghai 200032, China

#Authors contributed equally.

†Present address: Faculty of Pharmaceutical Sciences, Shenzhen University of Advanced Technology, Shenzhen 518107, China.

*Corresponding authors: Zhiwei Feng, Email: fengzhiwei@suat-sz.edu.cn. Yuanqiang Wang, Email : wangyqnn@cqut.edu.cn. Ying Xue, Email: xuey14@fudan.edu.cn. Li Meng , Email: 11172@jsmc.edu.cn

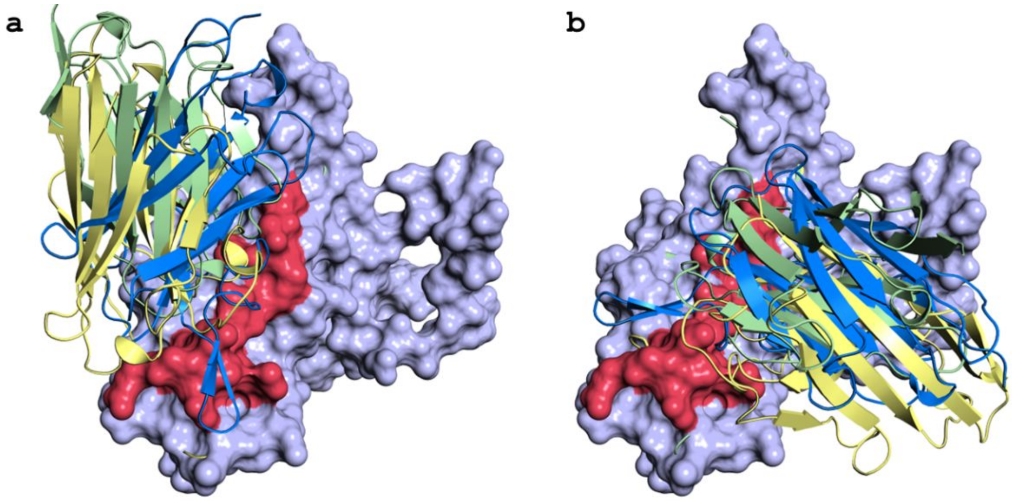

Figure S1. Structural alignment of the Trop-2–nanobody complexes.
(a) Alignment of the three nanobody–Trop-2 complexes in binding pose 1. (b) Alignment of the three nanobody–Trop-2 complexes in binding pose 2. Trop-2 is shown in purple, with the C-terminal cysteine-poor domain (CPD, Q237–Q252) highlighted in red. The nanobodies are represented as cartoon models: Nb60 in blue, Nb65 in yellow, and Nb108 in green. The structural alignment illustrates the highly similar binding orientations of the three nanobodies on the Trop-2 CPD region.

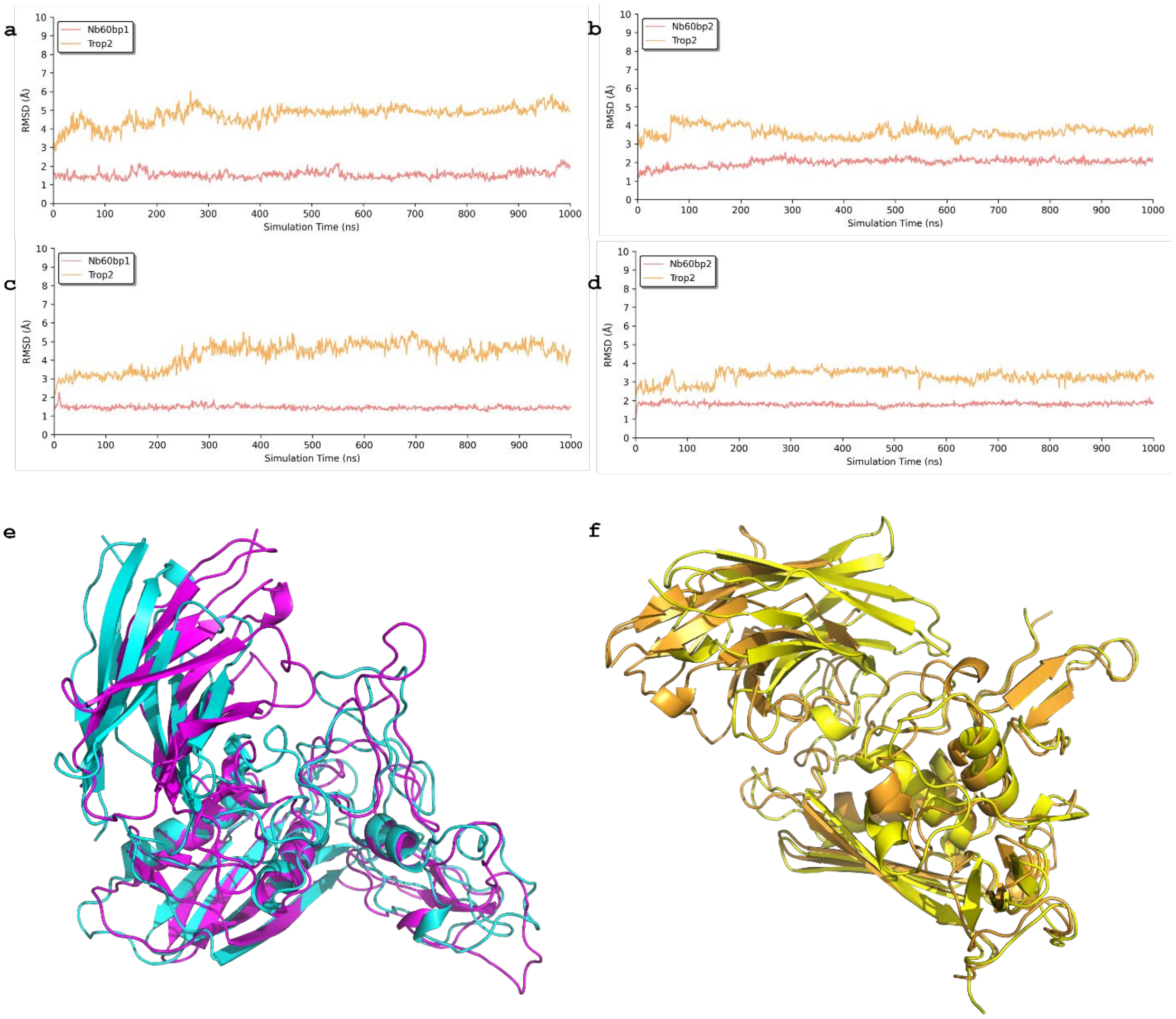

Figure S2. (A) Root Mean Square Deviation (RMSD) of Nb60 Binding Pose 1 from Round 2 Molecular Dynamics (MD) Simulation. (B) RMSD of Nb60 Binding Pose 1 from Round 3 MD Simulation. (C)RMSD of Nb60 Binding Pose 2 from Round 2 MD Simulation. (D) RMSD of Nb60 Binding Pose 2 from Round 3 MD Simulation. (E) Comparison of the average Nb60 Binding Pose 1 of the Round 2 MD Simulation (cyan) and Round 3 MD Simulation (magenta). (F) Comparison of the average Nb60 Binding Pose 2 of the Round 2 MD Simulation (yellow) and Round 3 MD Simulation (bright orange).

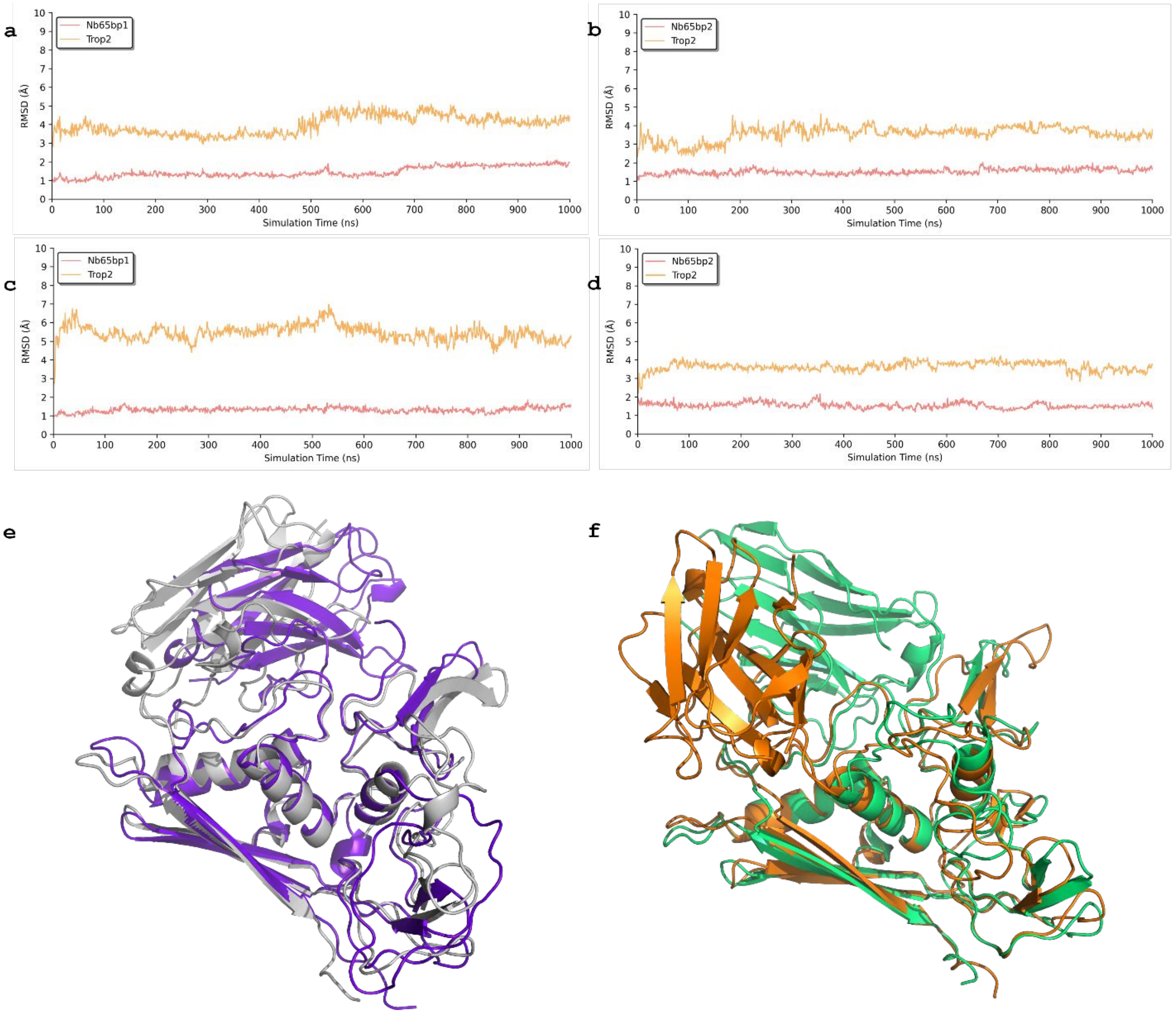

Figure S3. (A) Root Mean Square Deviation (RMSD) of Nb65 Binding Pose 1 from Round 2 Molecular Dynamics (MD) Simulation. (B) RMSD of Nb65 Binding Pose 1 from Round 3 MD Simulation. (C)RMSD of Nb65 Binding Pose 2 from Round 2 MD Simulation. (D) RMSD of Nb65 Binding Pose 2 from Round 3 MD Simulation. (E) Comparison of the average Nb65 Binding Pose 1 of the Round 2 MD Simulation (grey) and Round 3 MD Simulation (light purple). (F) Comparison of the average Nb65 Binding Pose 2 of the Round 2 MD Simulation (orange) and Round 3 MD Simulation (light green).

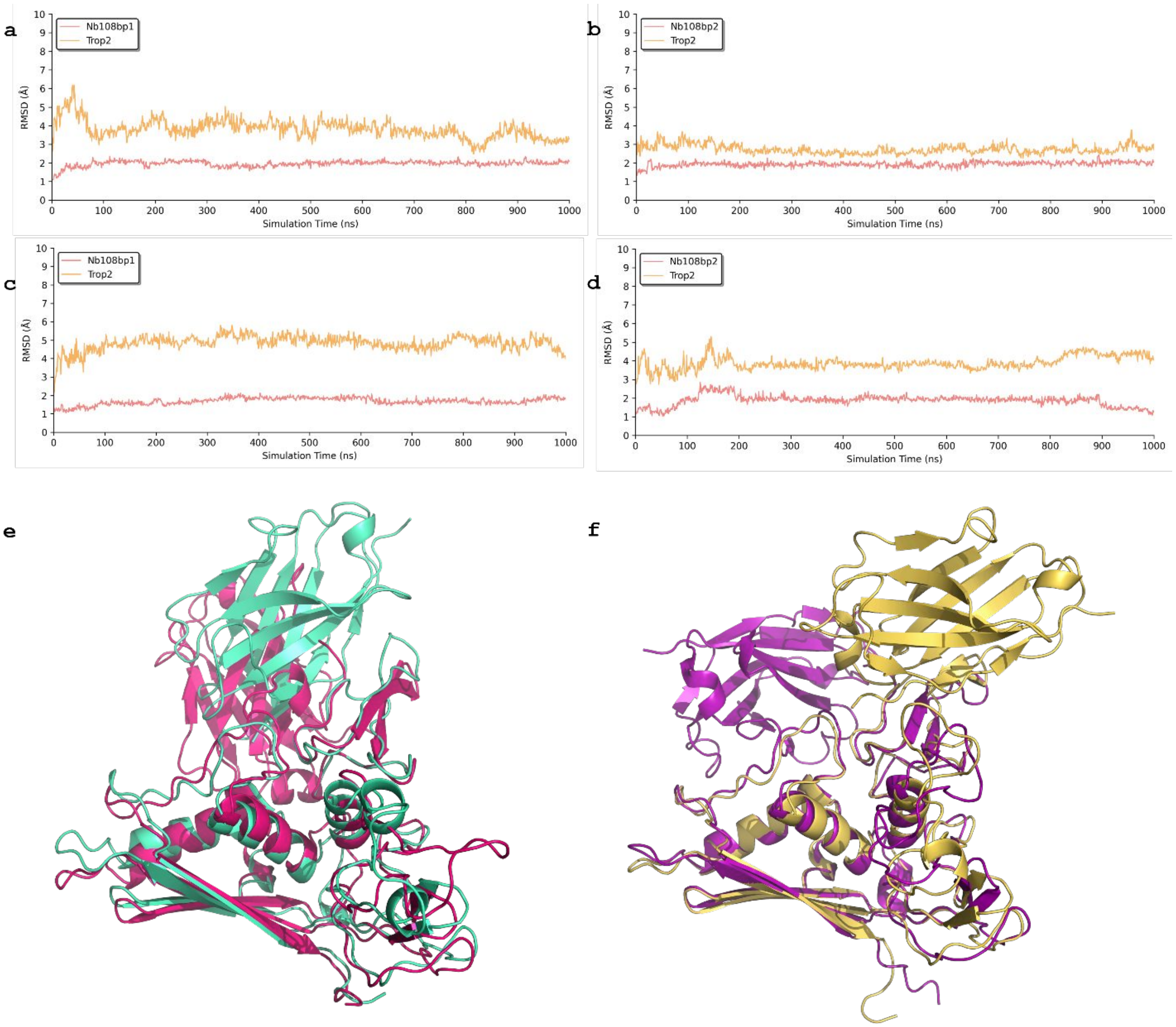

Figure S4. (A) Root Mean Square Deviation (RMSD) of Nb108 Binding Pose 1 from Round 2 Molecular Dynamics (MD) Simulation. (B) RMSD of Nb108 Binding Pose 1 from Round 3 MD Simulation. (C)RMSD of Nb108 Binding Pose 2 from Round 2 MD Simulation. (D) RMSD of Nb108 Binding Pose 2 from Round 3 MD Simulation. (E) Comparison of the average Nb108 Binding Pose 1 of the Round 2 MD Simulation (green cyan) and Round 3 MD Simulation (dark pink). (F) Comparison of the average Nb108 Binding Pose 2 of the Round 2 MD Simulation (gold) and Round 3 MD Simulation (purple).
